# Dynamic coronary CT Angiography-Estimated coronary flow in Non-Obstructive, Plaque-free coronary Arteries: Association with dyslipidemia and diabetes

**DOI:** 10.1016/j.ijcha.2022.101098

**Published:** 2022-08-12

**Authors:** Yukako Izoe, Michinobu Nagao, Kayoko Sato, Akiko Sakai, Kiyoe Ando, Miwa Kanai, Astushi Yamamoto, Shuji Sakai, Koichi Chida

**Affiliations:** a Graduate School of Medicine, Health Sciences, Division of Radiological Examination and Technology Tohoku University, Sendai City, Japan; b Department of Diagnostic Imaging & Nuclear Medicine, Tokyo Women’s Medical University, Tokyo, Japan; c Department of Cardiology, Tokyo Women’s Medical University, Tokyo, Japan

**Keywords:** Coronary CT angiography, Statin therapy, Dyslipidemia, Diabetes

## Abstract

**Rationale and Objectives:**

In this study, we implemented dynamic coronary CT angiography (CCTA) in order to estimate the coronary flow rate in morphologically normal coronary arteries as well as to identify factors affecting the coronary flow rate.

**Materials and Methods:**

We retrospectively enrolled 95 consecutively presenting patients without stenosis or plaque in their major coronary arteries on CCTA conducted with a 320-detector scanner (mean age, 57 years; 43 % men). Time-attenuation curves of the distal sites of the major coronary arteries and the aortic root were extracted from dynamic CCTA data. Coronary flow rate, an indicator of coronary blood flow, was calculated via a convolution-integration method integrating the two curves. Patients with dyslipidemia were divided according to the presence or absence of familial hypercholesterolemia (FH) as well as according to the receipt of statin therapy.

**Results:**

We found that the coronary flow rate was statistically significantly lower in statin-naïve patients with dyslipidemia (n = 27, 0.56 ± 0.10) than in patients without dyslipidemia (n = 32, 0.64 ± 0.10, p = 0.0013). In FH (n = 26), the coronary flow rate was statistically significantly lower in statin-naïve patients (n = 7, 0.65 ± 0.08) than in those taking statins (n = 19, 0.72 ± 0.10, p = 0.0221). Coronary flow rate likewise exhibited a statistically significant negative correlation with hemoglobin A1c (Pearson r, −0.437; p = 0.0003), but no correlation with other coronary risk factors. The coronary flow rate was statistically significantly lower in patients with diabetes (n = 14, 0.55 ± 0.10) than in those without diabetes (n = 81, 0.61 ± 0.11, p = 0.0461).

**Conclusion:**

We found a reduction in coronary flow rate in patients with statin-naive dyslipidemia and diabetes, even within morphologically normal coronary arteries.

## 1 Introduction

Recently, it has been reported that quantification of coronary flow using 320-row dynamic CCTA is useful for detecting myocardial ischemia and is likewise correlated with myocardial blood flow as estimated by ammonia positron emission tomography (PET) [1]. This is a unique technique that dynamically captures the contrast enhancement of coronary arteries *in vivo* and is expected to contribute information regarding coronary circulatory function (to a greater extent than to information regarding coronary artery morphology).

Atherosclerosis is a complex pathological process. Endothelial dysfunction, inflammation, and thrombogenesis all play a role in this process. Endothelial dysfunction is one of the first steps within the pathogenesis of atherosclerosis and can be caused by known risk factors for acute coronary syndrome and cardiovascular disorder [2]. In familial hypercholesterolemia (FH), the risk of CAD onset is extremely high, and angina pectoris and myocardial infarction frequently occur prematurely; these findings contrast with those of dyslipidemia occurring without a genetic background [3], [4]. Statin therapy has been shown to delay the age of onset of CAD [5] and to reduce the incidence of cardiovascular events in heterozygous familial hypercholesterolemia (HeFH) [6]. Statins protect the cardiovascular system not only by reducing low density lipoprotein (LDL) levels but also by decreasing LDL oxidation, stabilizing vulnerable atherosclerotic plaque, inhibiting vascular smooth muscle proliferation and endothelial dysfunction, and decreasing platelet activity [7], [8]. Moreover, statins inhibit other downstream products of the mevalonate pathway, causing so-called pleiotropic effects [9], [10], [11]. Effects on the vascular endothelium and on the activation of endothelial nitric oxide synthase are largely responsible for the role statins play in preventing CAD onset [12], [13], [14], [15], [16], [17].

In this study, we used dynamic CCTA to estimate the coronary flow rate in morphologically normal coronary arteries and to identify factors affecting the coronary flow rate.

## 2 Materials and methods

### 2.1 Study population

**Fig. 1:**
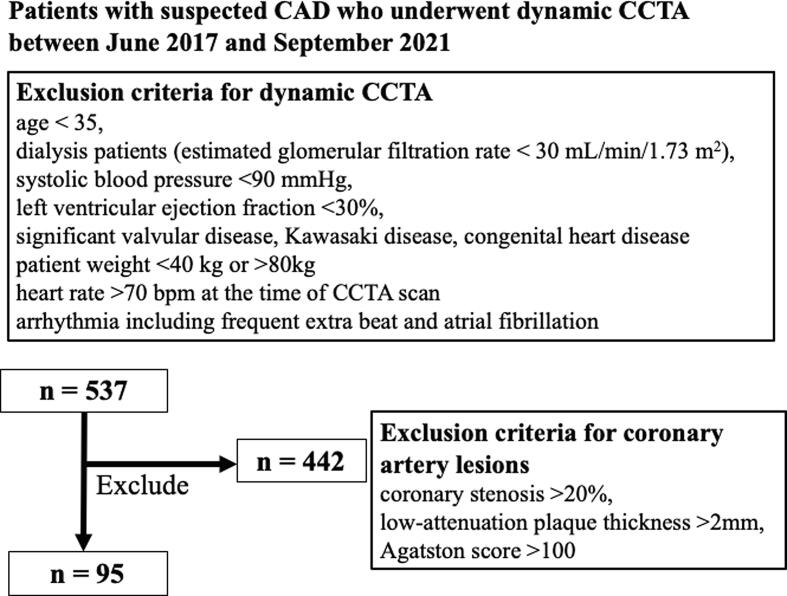
Flow diagram depicting patient enrollment procedures. CAD, coronary artery disease; CCTA, coronary computed tomography angiography.

We retrospectively evaluated 537 consecutively presenting patients with suspected CAD who presented at our University Hospital underwent dynamic CCTA between June 2017 and September 2021. Our University Hospital has more than 1,300 beds and provides highly advanced medical care, and the Department of Cardiology at this hospital has an outpatient clinic dedicated to FH. Patients are referred to this clinic from all over Japan. At our medical center, dynamic CCTA is performed in FH patients as a screening test for CAD. For all enrolled patients who underwent dynamic CCTA, written informed consent was obtained for their data to be used within a retrospective observational study titled “Development of a Method for Detecting Functional Ischemia by CCTA, Approval No 5517,” which was approved by the Ethics Committee of our university. This study complied with the principles of the Declaration of Helsinki and the guidelines of the ethics review board of our university. Ultimately, 95 patients with no more than 20 % stenosis in their major coronary arteries and a lack of low attenuation plaque more than 2 mm thick were enrolled in this study **(**Fig. 1**)** [18], [19], [20]. Other exclusion criteria were as follows: age < 35 years, dialysis patients (estimated glomerular filtration rate < 30 mL/min/1.73 m^2^), systolic blood pressure < 90 mmHg, severe left ventricular dysfunction (left ventricular ejection fraction < 30 %), significant valvular disease, Kawasaki disease, congenital heart disease, patient weights < 40 kg or > 80 kg, heart rate > 70 bpm, arrhythmia including frequent extra beat, and atrial fibrillation. Serum biochemical tests for dyslipidemia, diabetes, and renal dysfunction were performed within two weeks of undergoing the dynamic CCTA scan. For statin-naive patients, dyslipidemia was defined as a serum LDL of 140 mg/dL or [21]. FH was diagnosed based on current medical guidelines, including based on the results of genetic testing [22]. Any medical history of dyslipidemia, diabetes, and hypertension was extracted from patients’ medical records and was reviewed by the study authors. Dyslipidemia treatment was initiated with strong oral statins, with increased doses of statins and other dyslipidemia drugs given in cases of treatment resistance. Statin therapy was defined as taking a standard statin for at least six months. Patient’s characteristics at the time of dynamic CCTA are summarized in Table 1. Patients were considered statin-naïve if they had been off statins for at least six months prior to undergoing the dynamic CCTA scan. FH patient characteristics, including remnant-like particle cholesterol (RLP-C), malondialdehyde-modified LDL (MDA-LDL), and lipoprotein (a), are summarized in Table 2. Diabetes mellitus was defined as follows: receiving prescriptions of insulin or oral diabetic medication, and/or having a hemoglobin A1c (HbA1c) value of 6.5 % or higher (Table 3).

**Table 1:** Patient medical and demographic characteristics.

Number of subjects	95
Sex	
Male	41 (43.2)
Female	54 (56.8)
Age(years)	56.8 ± 11.4
Comorbidities	
Familial hypercholesterolemia	26 (27.4)
diabetes mellitus	14 (14.7)
Agatston score	
≥50 and < 100	9 (9.5)
<50	12 (12.6)
0	74 (77.9)

Values are presented as means ± standard deviations or as n (%).

**Table 2:** Patient characteristics in patients with familial hypercholesterolemia.

Number of subjects	26
Sex	
Male	11 (42.3)
Female	15 (57.7)
Age(years)	51.7 ± 10.8
Comorbidities	
Diabetes mellitus	4 (15.4)
Hypertension	6 (23.1)
Agatston score	
≥50 and < 100	4 (16.7)
<50	6 (25.0)
0	14 (58.3)
High-density lipoprotein cholesterol, mg/dL	69.8 ± 22.9
Low-density lipoprotein cholesterol, mg/dL	137.8 ± 63.0
Total cholesterol, mg/dL	235.5 ± 64.6
Remnant like particles cholesterol, mg/dL	8.3 ± 13.9
Malondialdehyde-modified LDL, U/L	128.5 ± 63.3
Lipo-protein (a), mg/dL	20.8 ± 18.1

Values are presented as means ± standard deviations or as n (%).

**Table 3:** Patient characteristics in patients with diabetes mellitus.

Number of subjects	14
Sex	
Male	11 (42.3)
Female	15 (57.7)
Age (years)	51.7 ± 10.8
Diabetes type	
Type 1	2 (14)
Type 2	12 (86)
Treatment	
Insulin	4 (16.7)
Oral diabetes medication	6 (25.0)
Untreated	14 (58.3)

Values are presented as means ± standard deviations or as n (%).

### 2.2 Dynamic coronary CT angiography

Dynamic CCTA was performed on all patients using a protocol utilizing dose-modulation and consisting of one series of standard CCTA (boost scan and low-dose dynamic imaging); more specifically, CCTA was conducted with a 320-row CT scanner (Aquilion One; Cannon Medical Systems Co., Tochigi, Japan) [1]. In preparation, intravenous or oral metoprolol was administered to patients with a heart rate of ≥ 65 beats/min. Immediately before image acquisition, all patients received sublingual nitroglycerin (0.2 mg). The test-bolus examination was performed using prospective electrocardiography (ECG)-gated axial scans at the ascending aorta in order to determine the optimal scan timing. Next, dynamic CCTA was continuously performed in mid-diastole for 8–12 cardiac cycles with prospective ECG-gating scans taken after a 10 s injection of the contrast medium (259 mg/kg, Iopamiron 370; Bayel Healthcare, Osaka, Japan). One scan of the dynamic CCTA was performed as a boost scan for standard CCTA at the peak phase of the ascending aorta. The scan parameters were as follows: gantry rotation time, 0.275 s; detector collimation, 320 × 0.5 mm; tube voltage, 100 kV; tube current, 80 mA at the dynamic scan, and auto radiation exposure reducing control at the boost scan with a field of view of 200 mm [23].

Morphological coronary stenosis and plaque was analyzed using dedicated software (Ziostation 2 Phyziodynamics; Ziosoft, Tokyo, Japan) based on a combination of transverse sections as well as automatically generated curved multiplanar reconstruction (MPR) images of the target vessels. Images were reconstructed from standard CCTA and were clinically interpreted by the consensus decision of two experienced radiologists and one cardiologist referencing the American Heart Association 15-segment model. Patients with coronary stenosis of > 20 %, with a low-attenuation plaque thickness of > 2 mm, and with Agatston scores of > 100 were excluded from the current study [18], [19], [20].

### 2.3 Calculation of the coronary flow rate

**Fig. 2:**
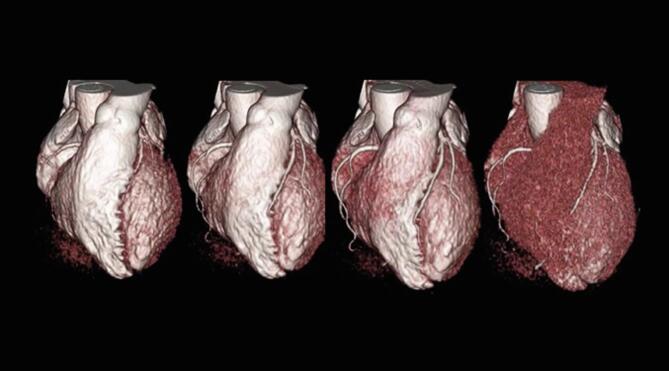
A volume rendering of dynamic coronary CT (computed tomography) angiography for a case of heterozygous familial hypercholesterolemia in a woman in her 40 s. This depiction shows the first pass of contrast media from the heart cavities into their normal coronary arteries. Herein, the flow is shown to be travelling from the left to the right over time.

**Fig. 3:**
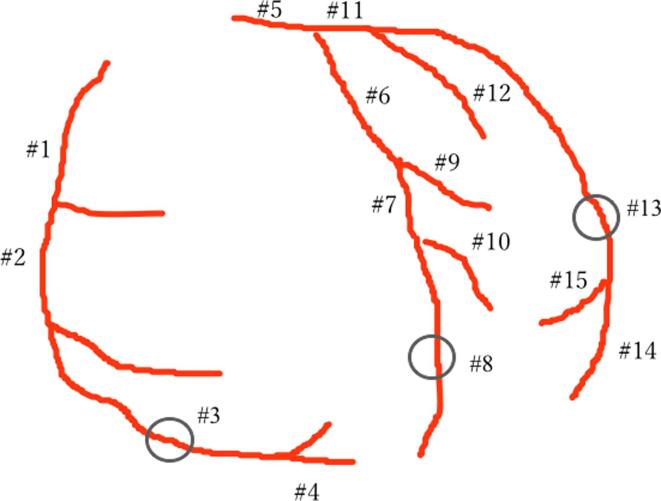
Coronary artery number according to American Heart Association (AHA) segment classification criteria (American Heart Association Committee Report). The spherical volume of interest (VOI) was drawn in the distal part of the coronary artery in the circled area (right coronary artery [RCA] segment # 3, left anterior descending artery [LAD], left coronary artery [LCX] # 13–14).

**Fig. 4:**
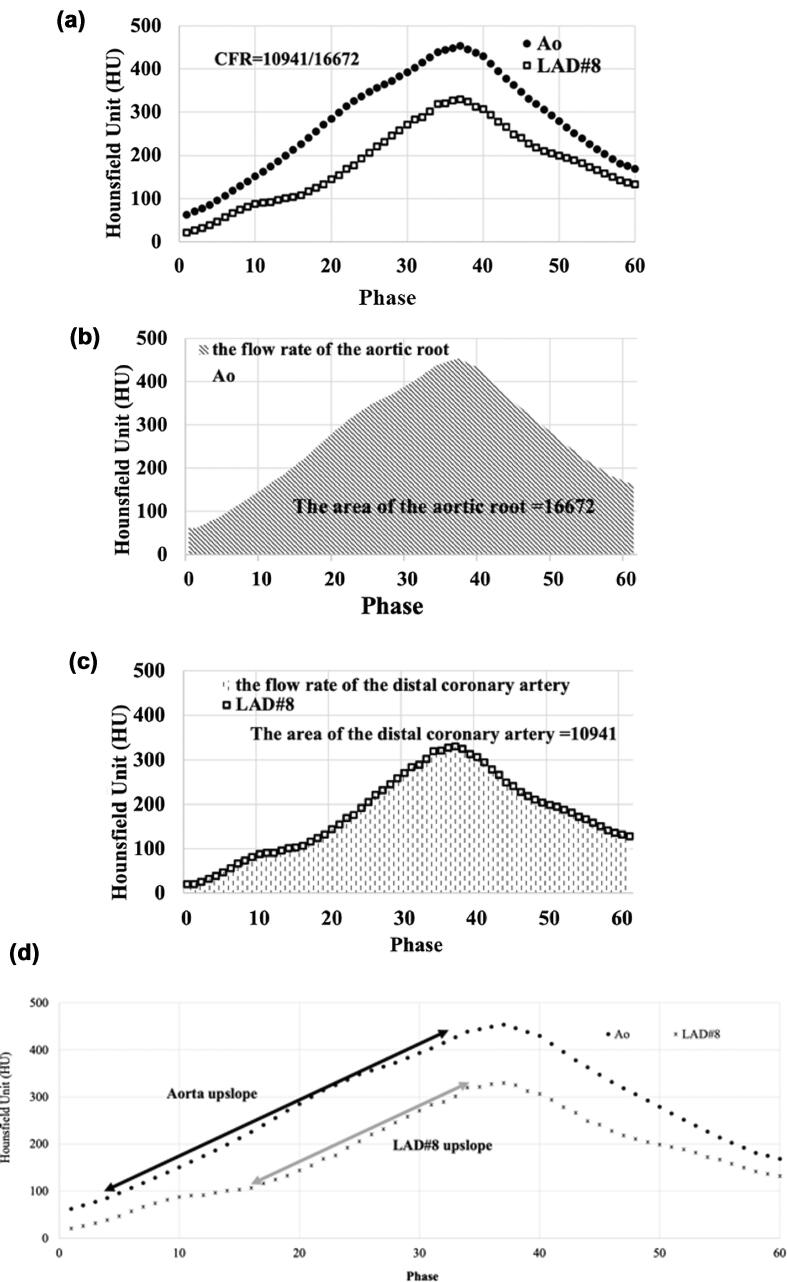
Calculation of the coronary flow rate (CFR). **A**. These graphs show time-density curves with regard to intravascular attenuation (HU; Hounsfield units) for the aortic root (Ao, ●) as well as for the distal portion of the left anterior descending artery (LAD #8) (□) throughout all phases of monitoring. The vertical axis represents the computed tomography (CT) value and the horizontal axis represents the time since the start of the dynamic scan. **B**. The area under the time-attenuation curve of the aortic root was calculated via integration in order to estimate the blood flow rate throughout the aortic root. **C**. Similarly, the area under the time-attenuation curve for LAD #8 was integrated to estimate the blood flow rate through LAD #8. The CFR was defined as the blood flow rate at LAD #8 divided by the blood flow rate at the aortic root (10,941/16,672 = 0.656). **D**. Calculation of the coronary flow index using the maximum slope method. The portions of the curve that linearize and increase in attenuation were extracted from the time-attenuation curves of the aorta and LAD#8, (black arrow, 3 to 33 phase; gray arrow, 16 to 34 phase). The attenuatuion gradient was then calculated. The coronary flow index was defined as the attenuation gradient of the aorta dived by that of LAD#8 (11.111/11.333 = 0.980).

Data from dynamic CCTA with 8 to 12 continuous cardiac cycles was transformed using dedicated software (Ziostation 2 Phyziodynamics; Ziosoft, Tokyo, Japan). The original data was interpolated according to neighboring phases for up to 40 to 60 dynamic image sets via motion coherence image processing (MCIP) (Fig. 2). [24] In the converted dynamic series, a spherical volume of interest (VOI) was drawn in the aortic root and distal portions of coronary arteries with a diameter of 3 mm (i.e., coronary segment #3 in the right coronary artery (RCA), coronary segment #8 in the left anterior descending artery (LAD), and coronary segments #13–14 in the left circumflex artery (LCX) (Fig. 3), as well as within the distal sites of the major branches, such as the diagonal, ramus, and obscure marginal). The VOI was set to avoid the arterial bend and was observed from various angles to ensure that it did not protrude from the vessel lumen. Once the VOI was activated, the coronary contour was automatically extracted by synchronization with the movement of the coronary artery. Each voxel in the VOI was tracked in all time phases. Variations in time and CT values (Hounsfield units) in the VOI were exported as numerical data, and the time-density curve of the VOI was created from the data generated within this procedure. The area under the time-attenuation curve represents the total amount of contrast media that had passed through the vessel, which is proportional to the blood flow rate. Individual flow rates can be standardized by dividing the flow rate of the distal coronary artery by that of the aortic root. Following this convolutional integration method, the coronary flow rate was defined as the area under the curve of the distal coronary artery divided by that of the aortic root. Coronary flow rates are dependent on accurate measurements of coronary artery attenuation throughout all dynamic phases. In the graph shown in Fig. 4, any deviating points prompted a check regarding whether the VOI did or did not deviate from the coronary contour in the movie reconstructed via motion coherence image processing. If the VOI deviated from the coronary contour, the setting was adjusted. The mean coronary flow rate of the three coronary arteries was evaluated as being representative of the patient’s overall coronary flow rate. However, there are known individual differences in coronary artery branching patterns, and approximately 20 % of patients have hypoplastic coronary arteries [25]. For this reason, coronary arteries with a myocardial dominant territory of less than 10 % of the total, as calculated using the Voronoi method, were excluded from the current analysis [26]. In addition, we analyzed time-attenuation curves for the evaluated coronary arteries based on the same dynamic CCTA data using the previously described maximum slope method (Fig. 4D) [1], [24].

### 2.4 Statistical analysis

Continuous data were expressed as means ± standard deviations. Testing of differences with regard to demographic data, clinical data, coronary flow rate, and other factors was conducted using the Kruskal-Wallis test along with Dunn’s multiple comparison test or the Mann-Whitney *U* test, as appropriate. Measures of the association among coronary flow rate and other factors were evaluated using Pearson’s coefficient analysis. All conducted statistical tests were two-sided. A *p*-value of < 0.05 was considered statistically significant. Interstudy variability was assessed via intraclass correlation coefficients (ICC). Agreement was considered excellent given an ICC of > 0.74, good given an ICC ranging from 0.60 to 0.74, fair given an ICC ranging from 0.40 to 0.59, and poor given an ICC of < 0.40. All analyses were performed using JMP statistical software (Version 9.0; JMP, Inc., Cary, NC, USA).

## 3 Results

### 3.1 Factors associated with coronary flow rate

The patient population was characterized as follows: more than half were female, approximately-one quarter were patients with FH, and dynamic CCTA showed that approximately 80 % of the enrolled patients had an Agatston score of 0 (that is, no calcification in the coronary arteries) (Table 1). In all 95 patients, body weight, heart rate, systolic blood pressure and diastolic blood pressure were 58.5 ± 8.9 kg, 57.0 ± 7.1 bpm, 122 ± 12 mmHg, 75 ± 8 mmHg, respectively. These hemodynamics indices did not differ in subgroups of patients with or without diabetes or with or without statin therapy. Coronary flow rates for RCA#3, LAD#8, and LCX#13 were 0.67 ± 0.11, 0.61 ± 0.16, and 0.67 ± 0.11, respectively. There were no statistically significant differences in the coronary flow rates of the three evaluated coronary arteries. Coronary flow rate showed a statistically significant negative correlation with HbA1c (Pearson r, −0.437; p = 0.0003). Moreover, coronary flow rate was not correlated with age (Pearson r, −0.057), LDL (Pearson r, −0.0293), total cholesterol (Pearson r, 0.137), triglycerides (Pearson r, −0.194), estimated glomerular filtration rate (Pearson r, −0.0438), uric acid levels (Pearson r, 0.122), hemoglobin (Pearson r, −0.052) or hematocrit (Pearson r, −0.052). There was no statistically significant difference in the coronary flow rate between patients with an Agatston score 0 and those with an Agatston score of 1 or higher (0.60 ± 0.11 vs 0.64 ± 0.09). Intraobserver ICC values with regard to the coronary flow ratio were as follows: 0.98 (95 % CI: 0.93 to 1.00) for RCA#3, 0.96 (95 % CI: 0.83 to 0.99) for LCX#13, and 0.95 (95 % CI: 0.80 to 1.00) for LAD#8. The interobserver ICC values were 0.98 (95 % CI: 0.93 to 1.00) for RCA#3, 0.95 (95 % CI: 0.80 to 1.00) for LCX#13, and 0.94 (95 % CI: 0.80 to 0.99) for LAD#8.

**Fig. 5:**
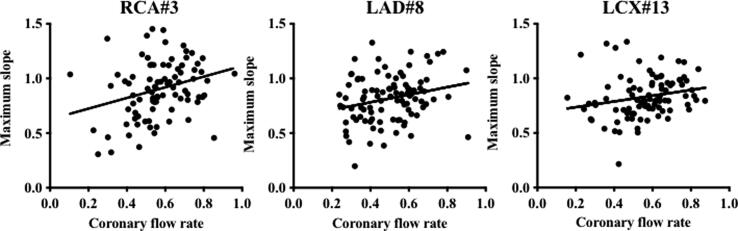
Scatter plots evaluating the coronary flow index according to the the maximum slope method as well as the coronary flow rate according the method proposed in the current study. For the three evaluated coronary arteries, we found a weak positive correlation between the two indices. The straight line reprsents the regression line showing the relationship between the two indices. Pearson’s correlation coefficients were as follows: 0.2819 for RCA#3, 0.2422 for LAD#8, and 0.2105 for LCX#13.

Using the previously reported maximum slope method [1], [24], we analyzed the time-attenuation curve of the evaluated coronary arteries based on the same dynamic CCTA data and presented the results of the coronary flow index calculation. The coronary flow indices for RCA#3, LAD#8, and LCX#13 were 0.90 ± 0.26, 0.82 ± 0.22, and 0.82 ± 0.20, respectively. The coronary flow index for RCA#3 was statistically significantly higher than for LAD#8 (p = 0.0158) and LCX#13 (p = 0.0258). The interobserver ICC was 0.85 (95 % CI: 0.83 to 0.87) for RCA#3, 0.83 (95 % CI: 0.80 to 0.86) for LCX#13, and 0.82 (95 % CI 0.80 to 0.87) for LAD#8. The Pearson correlation coefficients between the coronary flow rate as evaluated by this method and the coronary flow index as evaluated by the maximum slope method were as follows: 0.2819 for RCA#3 (p = 0.0074), 0.2422 for LAD#8 (p = 0.018), and 0.2105 for LCX#13 (p = 0.0465). There was a weak positive correlation between the two indices within the three coronary arteries (Fig. 5).

### 3.2 Relationships between coronary flow rate, dyslipidemia, and statin therapy

There were 32 patients without dyslipidemia and 63 patients with dyslipidemia enrolled in our study, of whom 36 were receiving statin therapy (Table 4). 90 % of the statins were strong statins, and 90 % of the latter patients had been taking these strong statins for>10 years. The coronary flow rate was statistically significantly lower in statin-naive patients with dyslipidemia (n = 27, 0.56 ± 0.10) than in patients without dyslipidemia (n = 32, 0.64 ± 0.10, p = 0.0013). There was no statistically significant difference in coronary flow rate between statin-treated patients with dyslipidemia (n = 36, 0.61 ± 0.12) and statin-naive patients with dyslipidemia**.**

**Table 4:** Patient characteristics according to hyperlipidemia and statin administration status.

	Patients without dyslipidemia	Statin-naïve dyslipidemia	Statin-treated dyslipidemia	P value
Number of subjects	32	27	36	0.461
Age (years)^a^	56.3 ± 12.1	55.5 ± 11.1	58.2 ± 11.2	
Sex^b^				
Male	15 (46.9)	15 (55.6)	11 (30.6)	0.122
Female	17 (53.1)	12 (44.4)	25 (69.4)	
High-density lipoprotein cholesterol, mg/dL^a^	68.1 ± 19.2	61.7 ± 17.9	70.3 ± 20.0	0.247
Low-density lipoprotein cholesterol, mg/dL^a^	113.0 ± 19.9	161.3 ± 43.0^c^	119.3 ± 40.0^d^	<0.0001*
Triglyceride, mg/dL^a^	120.4 ± 69.9	191.6 ± 218.9	130.1 ± 118.9	0.196
Total cholesterol, mg/dL^a^	196.2 ± 14.5	276.5 ± 42.5^c^	211.3 ± 51.1^d^	0.0003*
Hemoglobin A1c, %^a^	5.9 ± 0.3	6.1 ± 0.9	6.6 ± 1.5	0.140
Estimated glemerular filtration rate, mL/min^a^	73.3 ± 11.1	71.6 ± 12.7	70.8 ± 11.8	0.717
Uric acid, mg/dL^a^	5.1 ± 1.5	5.3 ± 1.6	5.2 ± 1.0	0.851

Values are presented as means ± standard deviations or as n (%). *p < 0.05.

a Evaluated through Kruskal-Wallis and Dunn’s multiple comparison tests.

b Evaluated through chi-squared tests.

c Statistically significant differences between the statin-naïve dyslipidemia patient group and in the group of patients without dyslipidemia.

d Statistically significant differences between the statin-naïve dyslipidemia patient group and the statin-treated dyslipidemia patient group.

**Fig. 6:**
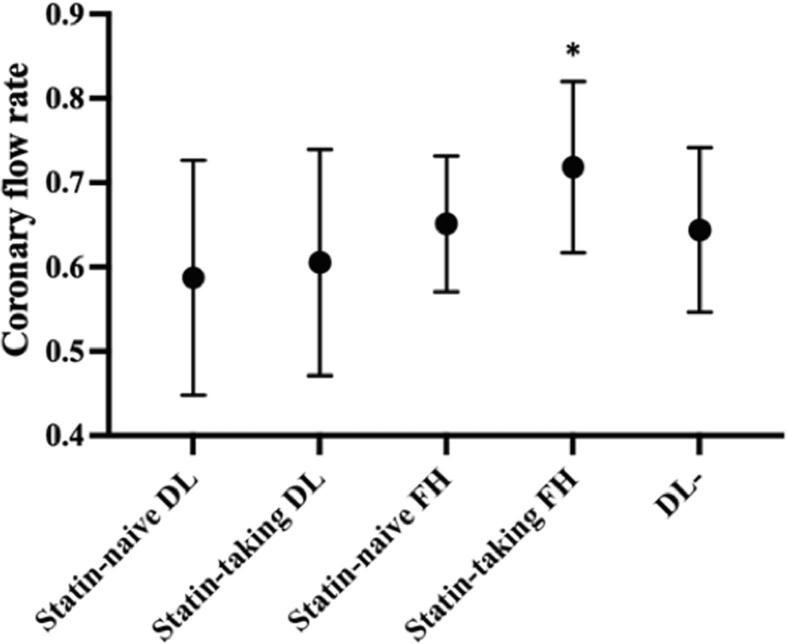
Coronary flow rate for dyslipidemic (DL) patients without familial hypercholesterolemia (FH), for patients with FH, and for those without DL. This graph depicts a comparison between statin-naïve and statin-treated patients. In FH, the coronary flow rate was statistically significantly lower for statin-naive FH patients than that for statin-treated FH patients. In DL patients without FH, there was no statistically significant difference between statin-naïve and statin-treated patients. *p < 0.05. Circles indicate mean values, and the upper and lower horizontal lines indicate standard deviations.

The dyslipidemia group included 26 patients with HeFH (mean age, 52 years; 42 % men). There were no homozygous FH patients. The coronary flow rate of the evaluated HeFH patients was 0.70 ± 0.10, which was statistically significantly higher than that of dyslipidemic patients without FH (n = 37, 0.60 ± 0.14, p = 0.0003). In HeFH patients, the coronary flow rate was statistically significantly lower in statin-naive patients (n = 7, 0.65 ± 0.08) than in statin-treated patients (n = 19, 0.72 ± 0.10*,* p = 0.0221). In dyslipidemic patients without FH, there was no statistically significant difference in coronary flow rate between statin-naïve and statin-treated patients **(**Fig. 6**)**.

### 3.3 Relationship between coronary flow rate and diabetes mellites

**Fig. 7:**
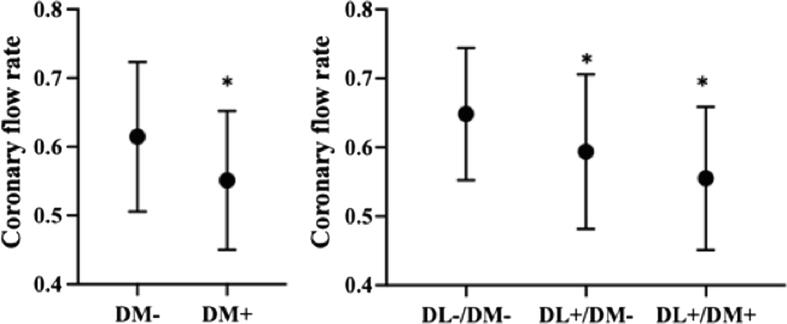
Comparisons of the coronary flow rate between patients with and without dyslipidemia (DL) and diabetes mellitus (DM). The coronary flow rate was statistically significantly lower for DM patients than that for non-DM patients (left). The coronary flow rate for patients that had neither DL nor DM was statistically significantly higher than in those with DL and without DM as well as in those with both DL and DM. There was no statistically significant difference between the latter two groups. *p < 0.05. Circles indicate mean values, and the upper and lower horizontal lines indicate standard deviations. Standard coronary CT (computed tomography) angiography showed no calcification or stenosis in the major coronary arteries (left, volume rendering; center, angiographic view). A curved multiplanar reconstruction image (right) of the left circumflex artery showed no low-attenuation plaque. A dynamic curved multiplanar reconstruction image of the left anterior descending artery. The intracoronary artery was contrasted almost uniformly and was then washed out. The flow is shown as travelling from the left to the right over time. Although the coronary arteries were morphologically unremarkable, the coronary flow rate (CFR) was reduced to 0.575. The mean CFR for patients without dyslipidemia was 0.70.

Of the 95 enrolled patients, 14 had diabetes mellitus, and their coronary flow rate was statistically significantly decreased as compared to the group (n = 81) without diabetes mellites (0.55 ± 0.10 vs 0.61 ± 0.11, p = 0.0461). The coronary flow rate for patients without both dyslipidemia and diabetes (n = 31, 0.65 ± 0.10) was statistically significantly higher than in patients with dyslipidemia and without diabetes (n = 50, 0.59 ± 0.11, p = 0.0127) and was likewise higher than in patients with both dyslipidemia and diabetes (n = 13, 0.55 ± 0.10, p = 0.0119*)*. There was no statistically significant difference between the latter two groups **(**Fig. 7**)**.

**Fig. 8:**
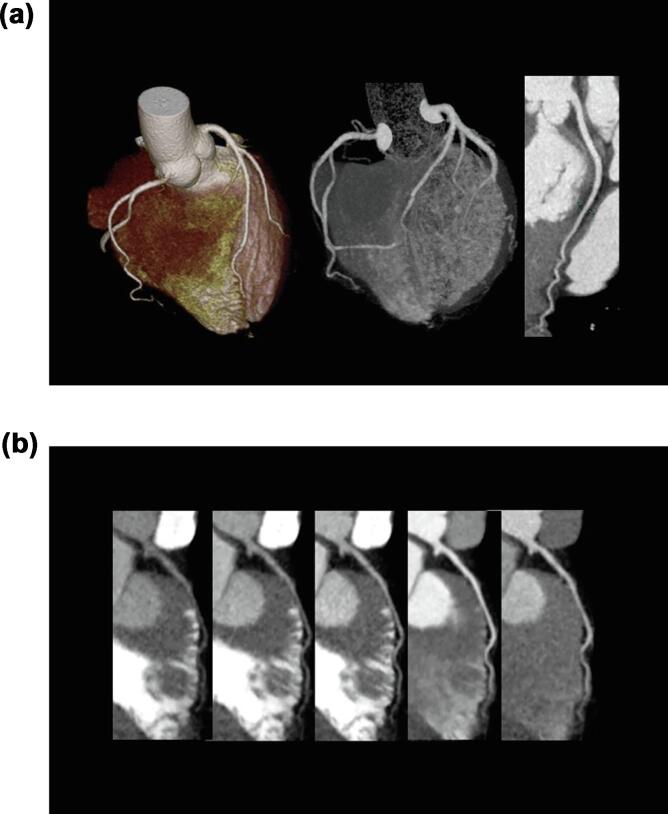
A woman in her 40s with statin-naive heterozygous familial hypercholesterolemia and a LDL (low-density lipoprotein) level of 260 mg/dL. **A.** Standard coronary CT (computed tomography) angiography showed no calcification or stenosis in the major coronary arteries (left, volume rendering; center, angiographic view). A curved multiplanar reconstruction image (right) of the left circumflex artery showed no low-attenuation plaque. **B.** A dynamic curved multiplanar reconstruction image of the left anterior descending artery. The intracoronary artery was contrasted almost uniformly and was then washed out. The flow is shown as travelling from the left to the right over time. Although the coronary arteries were morphologically unremarkable, the coronary flow rate (CFR) was reduced to 0.575. The mean CFR for patients without dyslipidemia was 0.70.

A typical case of heterozygous FH with reduced coronary flow rate in morphologically normal coronary arteries is depicted in Fig. 8**.**

## 4 Discussion

In the current study, we proposed a new method for quantifying coronary circulation using dynamic CCTA and found that the dynamic CCTA-estimated coronary flow rate was decreased in statin-naïve dyslipidemia patients and that this rate increased to the level of patients without dyslipidemia following statin therapy. As shown in Fig. 6, the coronary flow rate in HeFH patients on statin therapy was higher than that in patients without dyslipidemia. In these 19 patients with HeFH, LDL levels were controlled to an average of 122 mg/dL by treatment with strong statins; only 5 patients (26 %) had an LDL of 140 mg/dL or higher. Ten patients (53 %) who were refractory to statin therapy were given additional hyperlipidemic drugs. These strict treatments and follow-up protocols seem to have contributed to the high coronary flow ratio seen in HeFH patients within the current study. Our study population was limited to patients without coronary artery stenosis or atherosclerosis atheroma. In dyslipidemia, it is presumed that even morphologically normal coronary arteries suffer from hemodynamic disturbance. Thus, fluid mechanics based on coronary artery morphological changes are unlikely to be the cause of reduced coronary flow rates. Factors that may contribute to decreased coronary flow rate include increased blood viscosity due to high cholesterol, wall shear stress, and vascular endothelial dysfunction. Statins have been reported to regress and stabilize high-risk plaque within prior CCTA studies [27]. Additionally, statins have attracted attention for their pleiotropic effects on the vascular endothelium; these effects work to inhibit arteriosclerosis [28]. Moreover, statins inhibit prenylation of the Rac protein and the Rho protein, which leads to the increased expression of endothelium-derived nitric oxide synthetase. Moreover, nitric oxide production in the endothelium is increased and vasodilation is promoted with increased endothelium-derived nitric oxide synthetase expression [29], [30], [31]. We speculate that these mechanisms may be responsible for the increasing coronary flow rate seen following statin therapy.

Nitroglycerin (a premedication for dynamic CCTA) has been reported to dilate epicardial coronary arteries, thus resulting in a 40–50 % increase in coronary blood flow as compared to the resting state [32]. If there is vascular endothelial damage due to dyslipidemia, the coronary flow rate is reduced. This finding is consistent with impaired vasodilation. Interestingly, the coronary flow rate had a statistically significant negative correlation with HbA1c levels and was statistically significantly lower for patients with dyslipidemia and diabetes than in those without dyslipidemia and diabetes within the current study. Diabetes mellitus is the most common cause of vascular endothelial dysfunction [33]. High blood sugar levels can impair normal vasodilatation, and this effect is mediated by nitric oxide. We speculate that this mechanism may be the cause of the reduced coronary flow rate in diabetes mellitus. In contrast, there were no associations between age, coronary calcification scores, lipid metabolic function, or renal function in the current study. If a target population includes the elderly, cases with moderate to severe coronary calcification, and cases of reduced renal function, there may be correlations seen between the coronary flow rate and the above factors. With regard to dyslipidemia, statin status has been shown to have a stronger effect on the coronary flow rate than the severity of serum lipid levels. The efficacy of statins has also been reported in microvascular angina caused by vascular smooth muscle dysfunction [34]. Coronary flow rate, which is inversely proportional to downstream microvascular resistance, is a candidate for the detection of microvascular injury.

It has recently been reported that dynamic CCTA scanning can estimate functional myocardial ischemia [35] and can also estimate myocardial blood flow via ammonia PET [1]. The scan protocol used in this study, which combines standard CCTA with low-dose dynamic imaging using a 320-row scanner, does not increase radiation exposure as compared to conventional CCTA [23].

In previous reports [1], [24], the time-attenuation curve of coronary arteries was analyzed using the maximum slope method. However, this study adopted the convolutional integration method. As shown in Fig. 5, there was only a weak positive correlation between the two evaluated indices (Pearson r, 0.2105 to 0.2819), and the maximum slope method showed statistically significant differences among the three evaluated coronary arteries. When using the maximum slope method, individual variabilities in indices were larger than in the present method. Thus, corrections between coronary arteries may be necessary. The presently employed method calculates coronary flow rates from data within the entire curve, thus eliminating arbitrary factors and providing excellent reproducibility. Since FFR-CT is a simulation analysis that integrates information on coronary stenosis and vessel diameter, we think that it is impossible to detect functional abnormalities in morphologically normal arteries using this methodology. We note that morphologically normal arteries are the subject of this study, and that evaluating the coronary flow rate exceeds this limitation. More specifically, the utility of this methodology in detecting abnormalities within morphologically normal coronary arteries presents a major advantage over existing methods.

The main limitation of this study is that this is a retrospective, cross-sectional cohort study conducted at a single medical center. Ideally, the coronary flow rate should be compared individually before and after undergoing statin therapy. However, there are no current data on this topic because the benefits of performing multiple CCTA examinations on patients with morphologically normal coronary arteries are low, especially when considering the increasing radiation exposure and risk of contrast medium nephropathy associated with this methodology. We believe that a prospective study targeting patients with HeFH, who are thus at high risk for coronary artery disease, is highly necessary within future research efforts.

## 5 Conclusions

In the current study, we found a reduction in the coronary flow rate in patients with statin-naive dyslipidemia and diabetes, even when evaluating morphologically normal coronary arteries. Our findings inform future research directions and directly inform medical guidelines.
